# Objectively Measured Residential Environment and Self-Reported Health: A Multilevel Analysis of UK Census Data

**DOI:** 10.1371/journal.pone.0069045

**Published:** 2013-07-16

**Authors:** Frank Dunstan, David L. Fone, Myer Glickman, Stephen Palmer

**Affiliations:** 1 Institute of Primary Care and Public Health, School of Medicine, Cardiff University, Cardiff, United Kingdom; 2 Office for National Statistics, Newport, United Kingdom; University of Westminster, United Kingdom

## Abstract

Little is known about the association between health and the quality of the residential environment. What is known is often based on subjective assessments of the environment rather than on measurements by independent observers. The aim of this study, therefore, was to determine the association between self-reported general health and an objectively assessed measure of the residential environment. We studied over 30,000 residents aged 18 or over living in 777 neighbourhoods in south Wales. Built environment quality was measured by independent observers using a validated tool, the Residential Environment Assessment Tool (REAT), at unit postcode level. UK Census data on each resident, which included responses to a question which assessed self-reported general health, was linked to the REAT score. The Census data also contained detailed information on socio-economic and demographic characteristics of all respondents and was also linked to the Welsh Index of Multiple Deprivation. After adjusting for both the individual characteristics and area deprivation, respondents in the areas of poorest neighbourhood quality were more likely to report poor health compared to those living in areas of highest quality (OR 1.36, 95% confidence interval 1.22–1.49). The particular neighbourhood characteristics associated with poor health were physical incivilities and measures of how well the residents maintained their properties. Measures of green space were not associated with self-reported health. This is the first full population study to examine such associations and the results demonstrate the importance for health of the quality of the neighbourhood area in which people live and particularly the way in which residents behave towards their own and their neighbours’ property. A better understanding of causal pathways that allows the development of interventions to improve neighbourhood quality would offer significant potential health gains.

## Introduction

There is strong evidence that there are substantial area differences in health and wellbeing between residents of different neighbourhoods over and above differences due to socio-economic and cultural factors of individuals [1], [2]. Some of these differences are associated with variations in the social environment, but the effect of the physical environment is not well understood. A number of studies have considered how green outlook, incivilities, crime and noise might promote or harm mental health [3]–[6], with somewhat mixed results although generally finding an adverse effect of aspects such as incivilities and poor housing. Others have studied aspects which may impact on the level of physical activity including walkability of neighbourhoods, access to green spaces, street lighting and the fear of crime and road safety. For example Doyle et al [7] found that living in more walkable areas was associated with lower body mass index, though not with self-reported or physician-reported health. Some studies have considered access to the food environment [8] which may affect healthy eating, and others have investigated access to green spaces [9]. However few studies have considered associations between physical or mental health and the quality of the residential environment using an objective measure of quality.

Many studies assessing aspects of the quality of the physical environment have used a rating scale derived from questionnaires completed by residents. If these are completed at the same time as health and wellbeing data are collected, then there is a risk of same-source information bias [10]. Residents may be reluctant to rate their own areas as poor [11], but those ratings may also be influenced by personal circumstances. For example, people may rate their neighbourhoods more negatively when suffering from depression [12]. Studies have also varied greatly in their definitions of neighbourhoods. Many have used pragmatic definitions of administrative areas, such as those used in reporting census results or those defined for electoral purposes. These areas are not necessarily homogeneous with respect to social or physical environments, partly as they may be quite large, and their use may obscure causal factors that operate more locally down to street level. Others have attempted to use homogeneous areas [13], [14] but these were defined somewhat subjectively and cannot easily be generalised.

A number of attempts have been made to devise methods for measuring the quality of a neighbourhood by independent observers [13]–[17] but few studies have considered associations with health. Weich et al [17], in a study on two electoral wards in North London, showed a significant association between depression and specific aspects of house construction as well as the presence of graffiti, while Brown et al [15] showed significant associations between some neighbourhood characteristics and attachment to place and home. Burton et al [16] studied well-being, rather than health, in a small study of 200 people aged at least 65 and the results were not quantified.

In earlier work [18] we devised the Residential Environment Assessment Tool (REAT) to measure objectively and quantify the quality of the built environment at the smallest area-level of the UK full unit postcode. These postcodes cover small areas, with an average of 17 domestic households and about 35 residents, and give a more finely grained measure of place than many other areas used in such studies.

An earlier small study [19] using REAT gave inconclusive results on associations with mental health in a single town, partly because of the relatively small number of neighbourhoods studied. In this current study, REAT scores from a wider geographical area have been linked to self-reported health data on a whole population of more than 30,000 residents, using the UK 2001 Census [20]. The aim was to determine the association between self-reported general health and an objectively assessed measure of the residential environment. This is a much larger study than previous ones and uses a whole Census-enumerated population. This use of Census data allows adjustment for a wide range of socio-economic and demographic factors at individual and neighbourhood level, leading to a more effective assessment of the effect of the quality of the residential environment.

## Methods

### Setting

Wales, one of the constituent countries of the United Kingdom, has a 2001 Census population slightly in excess of 3 million, with an area of around 20,000 km^2^. The study took place in three geographically defined urban, formerly industrial areas in South Wales in which REAT scores had previously been measured. These were the study area of the Caerphilly Prospective Study [21] in Caerphilly county borough (CB) (n = 622 unit postcodes), the Upper Rhymney Valley (URV) (n = 104) sampled within the Caerphilly Health and Social Needs Study [22], and the study area of the Housing and Neighbourhoods and Health Study [19] in Neath Port Talbot (NPT) unitary authority (n = 51). REAT observations were made in 2001 for NPT and URV, and in 2005 for CB. These areas form part of two Unitary Authorities in South East Wales and contain many areas of material deprivation. In all three areas most of the population live in an urban environment but in towns with populations of up to 40,000, as opposed to large cities.

### Measure of Objective Neighbourhood Quality

Neighbourhood quality was measured at unit postcode level using a validated instrument, the Residential Environment Assessment Tool (REAT) [18]. The REAT score was devised as an objective neighbourhood measure, scored by independent observers, and is made up of 28 items recording aspects of the built environment covering the domains of physical nuisance and incivility, territorial functioning, defensible space, natural elements and miscellaneous other factors. These domains were selected on theoretical grounds as described in detail in [18]. Briefly, physical nuisance and incivilities, such as litter and vandalism and graffiti, may affect feelings of security and are associated with crime and the fear of crime [23]. This was measured by the prevalence of a number of items with negative connotations, including broken windows, vandalism, abandoned cars, stray dogs, general and dog litter. Territorial functioning [24] describes how well a neighbourhood is looked after by residents and was measured by evidence of property and garden maintenance, external beautification and neighbourhood watch signs. Defensible space, “a living residential environment which can be employed by inhabitants for the enhancement of their lives while providing security of their families, neighbours and friends” [25] was measured by the presence or absence of real or symbolic barriers such as hedges, fences and shrubbery impeding entry into a property, and property density. Natural environmental features, such as being able to see trees and greenery, are correlated with residential satisfaction [26]. A set of other questions that did not fall into the four categories described above was included; these focussed mainly on neighbourhood outlook such as view of industrial properties, derelict land, absence of recreational space, poorly maintained shared areas, undesirable parking and poor path condition and was in effect the obverse of presence of green features.

The 28 items were scored for each postcode by a trained observer who visited each postcode. When the instrument was first devised, pairs of observers scored each area independently; the very high degree of agreement found then led to a single observer being used in later work. Each item was given a score between 0 and 1, with clearly defined criteria for different scores, with all items scored so that higher scores represented lower neighbourhood quality. The individual items were assigned a weight of 1, 2 or 3 according to importance as determined by an independent survey of a random sample of a local authority’s citizens’ panel. The weighted scores were summed to give a score whose range was between 0 and 66, with lower scores indicating higher neighbourhood quality. Five subscales were also derived for the five categories described above. The scores were recoded into tertiles with approximately equal numbers of postcodes in each tertile. Tertiles (1 = highest quality, 3 = lowest quality) were treated as categorical variables in analysis to avoid assumptions of linearity. The five component scores were also recoded into tertiles specific to each category. This was the procedure used when the instrument was first devised [18] and the instrument was validated then against an individual-level questionnaire of over 1000 residents of the surveyed areas.

### Census Data

Individual Census records were extracted for all residents within the study postcodes and linked to REAT scores and to the Welsh Multiple Index of Deprivation (WIMD) [27] score, calculated for 2001 Census Lower Super Output Areas [28] within which the postcodes were located. Census data are highly confidential even without personal identifiers and the work was carried out inside the Office for National Statistics (ONS), who hold the data, following the signing of confidentiality agreements. The authors accessed the data using ONS’s secure virtual microdata laboratory (VML) facility [29].

The outcome measure was self-reported health with responses to the question:

*“Over the last twelve months would you say your health has on the whole been: Good? Fairly Good? Not Good?”.*

A binary variable was derived by combining the first two categories into a measure of ‘good health’, which was compared with ‘poor health’.

Census data were also available on age, gender, employment status, housing tenure, socio-economic status using the National Statistics Socio-economic Classification (NS-SEC) [30] and marital status. Employment status was divided into employed, seeking work, economically inactive and missing. Marital status was classed as single, married or with partner, separated/divorced or widowed. Social class was coded as professional, intermediate or manual, and other. Housing tenure was classed as rented or owner-occupied. Details of the census questions can be found at the ONS website [31].

### Statistical Analysis

Multilevel models were fitted, initially with three levels (individuals nested within households which were nested within postcodes). Since the mean number of respondents per household was only 1.8, a smaller number than is usually deemed appropriate for multilevel modelling, the household level was dropped from the analysis. A null model was first fitted to the binary outcome of good or poor health, with individuals nested within postcodes, and then the REAT scores were added and associations with self-reported health assessed. Further models were fitted adding individual-level covariates: age, gender, marital status, housing tenure, and employment status. A third set of models was fitted including WIMD standardised to a z score, and interaction terms between the REAT tertiles and the socio-economic characteristics. The models were fitted by MLwiN v2.02, using a second order marginal quasi-likelihood estimation method.

## Results

The study population comprised 31,442 residents aged 18 years or older in 777 postcodes.

Residents in different REAT tertiles differed significantly in socio-economic status and other demographic characteristics (Table 1). In tertile 1, with the highest neighbourhood quality, 25% were classed as professional compared to 18% and 12% in tertiles 2 and 3. Nine percent of people in REAT tertile 1 lived in rented houses compared to 24% in tertile 2 and 31% in tertile 3. Postcodes in Tertile 1 had lower percentages of single and economically inactive subjects, and had lower (less deprived) mean WIMD scores.

**Table 1:** Characteristics of residents of the tertiles of REAT scores.

	REAT tertile 1(highest quality)	REAT tertile 2(middle quality)	REAT tertile 3(lowest quality)	Total number
Sample size	8628	10977	11837	31442
Marital status				
Single	17%	22%	27%	6997
Married	66%	57%	53%	18181
Separated/divorced	8%	10%	12%	3254
Widowed	9%	10%	9%	3010
Housing tenure				
Owner occupier	91%	76%	69%	24436
Rented	9%	24%	31%	7006
NS-SEC				
Professional	25%	18%	12%	5493
Intermediate	15%	12%	11%	3859
Manual	23%	29%	35%	9401
Other	37%	41%	43%	12689
Employment status				
Employed	54%	49%	45%	15363
Seeking work	2%	3%	5%	1073
Inactive	34%	38%	42%	12046
Missing	9%	10%	9%	2960
Male gender	48.0%	47.7%	47.6%	15022
Age 18–44	37.5%	42.7%	47.3%	13512
Age 45–74	53.1%	47.1%	44.1%	14970
Age 75+	9.4%	10.3%	8.6%	2960
Mean (SD) WIMD	21.4 (14.7)	27.1 (15.7)	33.5 (15.4)	

Overall 19.6% of respondents said their health was poor, but this varied substantially by REAT score tertile. In each of the three regions the percentage with poor health increased with poor neighbourhood quality (Table 2). In the highest quality REAT tertile 15.2% reported poor health compared to 20.9% and 21.7% in the second and third tertiles respectively. Reported poor health was strongly associated with socio-economic characteristics. 31% of those in rented accommodation reported poor health compared to 16% in owner-occupied houses. There were also large differences by marital status and employment status.

**Table 2:** The percentage, with 95% confidence interval, of subjects in poor health, by socio-demographic and neighbourhood quality categories.

	Poor health %	95% CI	Total
REAT			
Tertile 1 (highest quality)	15.2	(14.2, 16.3)	8628
Tertile 2 (middle quality)	20.9	(19.8, 22.0)	10977
Tertile 3 (lowest quality)	21.8	(20.7, 22.8)	11837
Male	18.9	(18.0, 19.8)	15022
Female	20.4	(19.5, 21.2)	16420
Housing tenure			
Owner occupier	16.4	(15.8, 17.1)	24436
Rented	30.9	(29.4, 32.5)	7006
NS-SEC			
Professional	6.4	(5.5, 7.4)	5493
Intermediate	8.5	(7.3, 9.8)	3859
Manual	11.0	(10.1, 11.9)	9401
Other	35.2	(34.0, 36.4)	12689
Marital status			
Single	10.0	(9.1, 11.1)	6997
Married	19.6	(18.8, 20.5)	18181
Separated/divorced	24.7	(22.6, 26.9)	3254
widowed	36.7	(34.3, 39.2)	3010
Employment status			
Employed	4.1	(3.8, 4.5)	15363
Seeking work	7.4	(5.9, 9.1)	1073
Inactive	35.4	(34.6, 36.3)	12046
Missing	40.4	(38.7, 42.2)	2960

In a multilevel logistic regression model, in which individuals were nested within postcodes, but unadjusted for individual characteristics, the odds ratios for poor health by overall REAT score were 1.49 (95% CI 1.32 to1.70) for tertile 2, and 1.59 (95% CI 1.40 to1.80) for tertile 3, both compared to tertile 1 with the highest neighbourhood quality.(Table 3). For the five subscales within REAT, odds ratios were significantly raised for tertiles 2 and 3 relative to tertile 1 for physical incivilities and territorial functioning but they were significantly lower in those tertiles for the miscellaneous category (Table 3).

**Table 3:** Odds ratios, and 95% confidence intervals, of poor health for tertiles of the REAT score and its components, with the tertile of highest quality as reference, both unadjusted and adjusted (1) for individual-level covariates of age, gender, housing tenure, marital status and employment status and (2) for individual-level covariates and area deprivation.

	Unadjusted OR (95% CI)		Adjusted OR^1^ (95% CI)		Adjusted OR^2^(95% CI)	
Variable	Tertile 2 (middle quality)	Tertile 3 (highest quality)	Tertile 2 (middle quality)	Tertile 3 (highest quality)	Tertile 2 (middle quality)	Tertile 3 (highest quality)
REAT	1.49(1.32, 1.70)	1.59(1.40, 1.80)	1.42(1.29, 1.57)	1.47(1.32, 1.63)	1.36(1.24, 1.51)	1.36(1.22, 1.49)
Physical incivilities	1.40(1.21, 1.60)	1.57(1.36, 1.82)	1.34(1.19, 1.50)	1.41(1.26, 1.59)	1.34(1.20, 1.49)	1.30(1.16, 1.46)
Territorial functioning	1.50(1.32, 1.70)	1.83(1.63, 2.05)	1.40(1.26, 1.57)	1.59(1.45, 1.75)	1.33(1.20, 1.47)	1.47(1.33, 1.61)
Defensible space	1.07(0.94, 1.23)	1.11(0.98, 1.26)	1.18(1.07, 1.31)	1.17(1.06, 1.29)	1.18(1.07, 1.30)	1.18(1.08, 1.30)
Natural elements	0.90(0.79, 1.03)	0.96(0.84, 1.11(	1.09(0.99, 1.21)	1.06(0.95, 1.19)	1.01(0.91, 1.12)	1.03(0.93, 1.14)
Miscellaneous	0.83(0.74, 0.93)	0.80(0.70, 0.91)	0.94(0.85, 1.03)	0.93(0.84, 1.03)	0.97(0.89, 1.07)	1.00(0.91, 1.11)

After adjusting for age, gender, marital status, housing tenure and employment status of the individuals resident within postcode areas, self-reported poor health was still associated with poorer REAT scores (Table 3); the NS-SEC classification did not significantly improve the model. Odds ratios were significantly greater than 1 for the overall score and for the sub-components of physical incivilities, territorial functioning, and defensible space.

When the deprivation measure, WIMD, for postcode areas was added to the model it was associated with poor health with an odds ratio of 1.16 (1.12–1.21) for a change of 1 standard deviation (SD) in the WIMD score, but the associations with REAT were essentially unaltered, with the odds of poor health being 37% higher in neighbourhoods in the second and 36% in the third REAT tertiles than in the first tertile (Table 3). Odds ratios were also significantly greater than 1 for physical incivilities, territorial function and defensible space; for natural elements and miscellaneous features they were not significantly different from 1. The postcode random effects had a standard deviation of approximately 0.27, after adjustment, so that postcodes differing by two standard deviations in their random effects would have the probability of poor health differing by a factor of approximately 1.7, suggesting a considerable postcode effect.

Interactions between age, gender, employment status and REAT tertiles were analysed. We found that the association between poor health and REAT tertiles was less strong in women who were economically inactive, but that this effect decreased with increasing age. No other significant interactions were found and the effect on estimates of the odds ratios associated with REAT for other groups was small. Because many of the REAT scores were measured later than the Census date we included terms in the model to allow for differences in the effect of the REAT tertiles between these postcodes, but the results were non-significant and effect sizes small, suggesting the neighbourhood quality effects did not vary with area.

## Discussion

Health policy, both nationally [32], [33] and internationally [34], has identified the need to consider the role of the built environment in population health and health inequalities, but research to date has not given a consistent picture. This could be due to methodological issues such as variation in the way areas are defined and lack of objective unbiased measures of the quality of the built environment. Insights into the way in which the built environment may affect health could have implications for the design of new neighbourhoods or for the regeneration of existing ones and so the problem is of considerable public health importance. It is therefore important to conduct rigorous studies and this study is the first of which we are aware that has investigated associations between general health in a large census population and an objective validated measure of neighbourhood quality. We used data from a self-rated general health question included in the 2001 UK Census [20] for postcodes for which we measured neighbourhood quality using REAT scores. Poor general health has been shown to be strongly predictive of mortality rate [35] and of health care utilization [36]. Poor general health is also strongly linked to lower socio-economic status and so the associations we found with WIMD, housing tenure, employment status and marital status are as expected [37].

We found that the risk of poor general health was substantially higher in areas of low neighbourhood quality compared to those of high neighbourhood quality, even after adjusting for both individual socio-economic factors and also for area deprivation; the odds ratio of 1.36 translates into an increased risk of almost 30%. We did not find a clear dose response relationship; the middle and worst tertiles had very similar excess risks of poor health, suggesting the possibility of a threshold effect for aspects of neighbourhood quality.

In the first study of associations between REAT and mental health in one Local Authority in Wales, REAT was not clearly related to mental health as measured by the 12-item General Health Questionnaire [19], but the power of the study was relatively low with only 51 postcodes. However, an adaptation of REAT in a larger study [38] in Santiago, Chile revealed a significant association between measures of the built environment and mental health, as assessed by the Revised Clinical Interview Schedule [39]. They constructed different domains from those used here but those which had significant associations corresponded approximately to physical incivilities; they also did not find an association with the presence of green areas.

The associations we have found between neighbourhood quality and general self-reported health are surprisingly large. Burton et al [16] have emphasised that an important public health goal of this type of research is to identify causal factors that may be modified by urban planning and architectural design to improve population health and wellbeing. The sub-components that we found to be important are mainly concerned with incivilities and pride taken in a neighbourhood by its residents. Our findings add to the growing body of evidence that social incivilities and crime, or the fear of crime, have a strong influence on mental health and inhibit physical activity [40]. These factors might be more amenable to change than the basic structure of a neighbourhood, whether directly by interventions to address the physical incivilities or through enhancing social cohesion.

In common with Araya et al [38] we found no evidence of any associations between the features of the natural environment and poor health. It is possible that our measurement tool omitted key features. It focussed on whether there was green space in the immediate area of the postcode and so the presence of a park a short distance away would not be recorded. A study [26] in Australia reported an association between both physical and mental health and the perceived greenness of a neighbourhood, defined as a much larger area than here. This association became non-significant when adjusted for recreational walking, suggesting that it may be the presence of areas suitable for walking, such as parks, in a rather larger neighbouring area that might be important. Other studies [41], [42] have shown associations between physical or mental health and the presence of green space in the immediately surrounding area. There is a need for further work to try to identify the causal mechanisms involved.

For studies of the local environment, the choice of small areas, or neighbourhoods, is clearly important. For practical reasons, administratively-defined areas have generally been used, although there is no guarantee that these represent social communities. Areas used vary considerably in size and hence in heterogeneity. In the UK the Office for National Statistics uses a hierarchy of output areas for reporting census results and these were constructed to ensure a reasonable degree of social homogeneity, to have a compact shape and to preserve natural boundaries such as rivers or large roads. The level in the hierarchy most widely used is the Lower Super Output Area (LSOA), which has a minimum of 1000 residents and an average of approximately 1500. Within an LSOA there can be considerable variation in several aspects of a neighbourhood and we preferred to use smaller units. In the US census tracts have been used but these are larger again, varying between 1500 and 8000 persons. A recent paper from Canada [43] moved away from administratively-defined areas to use historical, socio-economic and perceptual viewpoints but produced areas with an average population of approximately 5000 persons. When assessing the presence of facilities near an individual, a quite different approach has been used by some investigators. Geographical Information Systems have been used to define a buffer around each residence, for example with a radius of 1 km, and counts are made of the number of food outlets, for example, within this. While this is a meaningful measure in that context, it appears less appropriate for neighbourhood quality.

### Strengths and Limitations

Many studies of neighbourhoods are based on survey data collected from residents covering both self-reported health and neighbourhood quality and therefore may be subject to same-source bias. We used an independently derived accepted UK measure of general health for areas for which we had objectively assessed neighbourhood quality data. As far as we are aware this is the first study to accomplish this.

The measure of neighbourhood quality, REAT, used independently trained observers to rate the postcode areas using features identified within an architectural sciences framework and based on an extensive review of the literature [18]. Other measurement instruments have been developed to move away from reliance upon individuals’ self reported perceptions of neighbourhood quality [13], [16], [44], [45]. For example, Weich et al [13] developed a site survey instrument published in 2001 which characterised built form, housing, access and other aspects including features obviously related to quality of neighbourhood such as disused or derelict buildings, evidence of vandalism and graffiti, and territorial functioning. Burton et al [16] refined this into the Neighbourhood Design Characteristics Checklist of 25 items to study built environment and healthy ageing. However, few data have been published to date on the utility of such tools in identifying remedial area factors in the causal pathway to health. Our study is by far the largest small area assessment that we have identified, studying objective measures of 777 neighbourhoods.

Many studies of neighbourhood effects that appropriately use multilevel modelling to separate out individual from contextual factors are nevertheless underpowered to identify small but important effects. In this study we have a virtually complete population sample of over 30,000 adults living in 777 neighbourhoods that were characterised independently using a validated measurement instrument. Issues of response bias that affect many studies with low response rates were avoided in this study.

One potential weakness of the study was that the majority of the REAT scores, those in the borough of Caerphilly, were measured four years after the general health question was recorded in the 2001 census. We have used the later REAT scores as proxies for the scores in 2001 but this could lead to inaccuracies if neighbourhoods changed substantially over that period. This has the potential to introduce bias [46] but local knowledge suggests this is unlikely. The communities comprising the Caerphilly borough that we studied are long established, and the area levels of socio-economic deprivation and poor housing quality have unfortunately been highly resistant to change [47]. Interaction terms between the REAT tertiles and the three study areas were included in the model and were non-significant, suggesting that the associations between health and neighbourhood were not different in those whose REAT scores were measured later; while this does not exclude the possibility of a temporal effect, it supports the inclusion of all the areas in a single analysis.

Our study is limited in that it uses a single measure of self-reported general health [48] analysed as a binary variable. Validation of this census measure has shown a strong correlation with all-cause mortality but this relationship may be attenuated under some circumstances [49], [50].

The associations found in this study are cross-sectional and therefore cannot demonstrate a causal link. It is conceivable that people with poor self-reported health are more likely to move to areas of low neighbourhood quality, or less likely to move out to areas of higher quality, and therefore the direction of causality could in theory be the reverse of that hypothesised. However, given the associations persist after adjusting for individual socio-economic factors, we think this explanation is unlikely, though we acknowledge that there are many socio-economic factors that are not fully captured by the census information. These adjustments were based on the data available in the Census and included a variety of factors known to be related to health, at both individual and area levels. It is possible, however, that there is residual confounding due to unmeasured confounders; this is always a potential problem with observational studies.

### Conclusions

In this large complete population study using independently derived objective measures of neighbourhood quality and general health, people living in the worst two-thirds of neighbourhoods, as measured by an objective quality score, have a nearly 30% greater risk of poor health, independent of individual and area-level socio-economic factors. We found that poor self-reported health was strongly associated with physical incivilities, territorial functioning and defensible space but not features of the natural environment, stressing the role of social pathways in generating area inequalities in health. More research is needed to try to elucidate the causal pathways so that interventions can be devised to reduce these environmental effects.
